# Effects of various surgical procedures on biochemical parameters of Nigerian dogs and their clinical implications

**DOI:** 10.14202/vetworld.2018.909-914

**Published:** 2018-07-08

**Authors:** Aboh Iku Kisani, Terzungwe Tughgba, Abdullahi Teleh Elsa

**Affiliations:** Department of Veterinary Surgery and Theriogenology, College of Veterinary Medicine, Federal University of Agriculture, P.M.B. 2373, Makurdi, Benue State, Nigeria

**Keywords:** anesthesia, cortisol, stress response, surgery

## Abstract

**Aim:**

The importance of physical and psychological stress caused by pain during surgery cannot be overemphasized. The aim of this study was to assess the effects of ovariohysterectomy (OVH), gastrotomy (GST), and intestinal resection and anastomosis (IRA) on biochemical parameters of Nigerian dogs anesthetized with the xylazine-propofol combination.

**Materials and Methods:**

A total of 12 dogs were randomly divided into three groups of four each. The animals were treated with xylazine and propofol anesthetics for OVH, GST, and IRA in Groups 1-3, respectively. Blood samples were collected at 0 h, 2, 24, 48, 72, 96, 120, and 144 h postsurgery for determination of cortisol (CORT), glucose (GLU), total protein (TP), albumin (ALB), globulin (GL), and ALB/GL ratio.

**Results:**

There were significant increases (p<0.05) in mean CORT concentrations 2 h postsurgery in the three groups and at 24 h in Group 3 and 96 h in Groups 1 and 3, respectively. GLU concentrations increased significantly (p<0.05) in the three groups at 2 h postsurgery. The mean protein concentrations in Groups 2 and 3 decreased significantly (p<0.05) at 2h and 24 h and 72 h, 96 h, 120 h, and 144 h in Group 3, respectively. There was significant decrease (p<0.05) in the mean ALB concentrations at 2 h, 24 h, 72 h, 96 h, 120 h, and 144 h postsurgery. There was a significant difference (p<0.05) in the mean GL concentrations in Group 3 at 24 h, 72 h, 96 h, and 144 h. All other parameters were not significantly different (p>0.05) in comparison with the control.

**Conclusion:**

Xylazine-propofol combination could decrease humoral immune status and increase serum GLU level invariably portending a high risk of diabetes in the vulnerable dogs.

## Introduction

Anesthetics such as thiopentone and ketamine are used for induction and maintenance of anesthesia in both elective and major surgery in the Nigerian local dogs. The pharmacokinetics and pharmacodynamics of the drugs have also been reported in the dogs with relatively long and short time effects [1,2]. The dogs are always kept for security and company. However, the effects of surgical procedures on biochemical parameters of the dogs using general anesthetics have not been determined.

Ovariohysterectomy (OVH), gastrotomy (GST), and intestinal resection and anastomosis (IRA) are surgical procedures that are commonly performed in practice. They are performed on the elective and emergency basis and are capable of eliciting variable degrees of surgical stress response which if not allayed by the use of anesthetics and analgesics can result in untoward effects [3-5].

Stress and pain caused by surgery and the risk associated with anesthesia and analgesia are quite challenging to both clinicians and the patients [6,7]. Surgical stress occurs, before, during, and after an operative procedure. It arises from psychological stress, tissue injury, and alterations in circulation, anesthetic agents, and postoperative complications including sepsis [8]. Surgical stress response involves stimulation of the sympathoadrenal medullary and the hypothalamic-pituitary-adrenal axis [9-13]. Their activation causes endocrine and immunomodulatory changes after trauma [14-17]. The degree of physiological response is proportional to the magnitude of injury with increased demands on organ function [18-22].

Response to surgical stress is a compensatory mechanism that prevents secondary damage and increases the availability of substrates required by essential organs and healing tissues [23]. However, if the stress response is prolonged, it results in longer hospitalization [10,18,24-26]. Therefore, alleviating prolonged pain and stress in surgical patients is important for animal health [27-29].

However, there is a paucity of information on effects of various surgical procedures on biochemical parameters of the Nigerian dogs. Furthermore, the implications of biochemical changes after surgery have not been very much recognized. Propofol is an anesthetic agent used in veterinary surgery for induction and maintenance of anesthesia. It causes smooth induction, rapid recovery with short half-life, and insignificant postoperative complications [30]. It has anti-stress and antioxidant properties [31]. Xylazine is an alpha2-adrenoceptor agonist that is used for premedication, sedation, and analgesia in small animal practice [32]. It has marked anesthetic effect [33].

The aim of this study was to assess the effects of ovariohysterectomy (OVH), gastrotomy (GST), and intestinal resection and anastomosis (IRA) on biochemical parameters of Nigerian dogs anesthetized with the xylazine-propofol combination.

## Materials and Methods

### Ethical approval

This study was conducted in line with NIH [34] Guidelines and the recommendation of the Ethical Committee of College of Veterinary Medicine, University of Agriculture, Makurdi, Nigeria.

### Experimental animals

A total of 12 adult Nigerian local dogs were used for this study. The dogs were housed in kennels at Veterinary Teaching Hospital, University of Agriculture, Makurdi, Nigeria and stabilized for 4 weeks. They were fed commercial dog food (Bingo^®^) manufactured by Grand cereal Nigerian Ltd, Jos, Nigeria. Water was provided *ad libitum*. Four animals per group were used according to the recommendation of Saganuwan [35].

### Drugs

Chlorhexidine gluconate (Saro life care limited, Nigeria), normal saline (Dana pharmaceutical, Nigeria), atropine sulfate (Jiagsu Huayang Pharmaceutical, China) and xylazine (XYL-M2, VMD, Belgium) and propofol (Frensenius Kabi AB SE-75174 Uppsala, Sweden) were obtained from Vincal pharmaceutical shop in Makurdi, Nigeria.

### Surgical materials

Chromic catgut (Anhu Kangning Industrial Group, Co. Ltd., China) and Nylon manufactured (Pal Pharmaceutical, China) were purchased from Vincal Pharmaceutical shop in Makurdi, Nigeria.

### Preoperative preparation

Four each of the dogs were used for OVH, IRA, and GST. The ventral abdominal area of each animal was shaved, scrubbed, and aseptically prepared using 0.05% chlorhexidine gluconate (purit^®^, Saro lifecare limited, Nigeria). The animals were infused with normal saline 1 mg/10 ml/h.

The dogs were treated before surgery with intravenous atropine sulfate (0.04 mg/kg) and xylazine (2 mg/kg, intramuscularly). Anesthesia was induced with propofol (6 mg/kg intravenously).

### Surgical procedures

#### OVH

The incision was made on the skin of the ventral midline and the subcutaneous tissue to expose the linea alba. After that, a stab incision was made on the linea alba with scissors to explore the abdomen. The urinary bladder was reflected to expose the uterine body. The right and the left uterine horns and the ovaries were located. The ovarian pedicle was double ligated using chromic catgut (size 2-0), and the two ovaries and uterus were removed. The abdominal and skin incision were stitched using chromic catgut and nylon (sizes 2-0), respectively.

### IRA

A ventral midline abdominal incision was made and the intestinal tract exteriorized. About 10 cm of the small intestinal tract was resected beginning from a point 7 cm from the duodenojejunal flexure (Treitz ligament). The residual intestinal tract was sutured using end-to-end anastomosis with chromic catgut (size 2-0).

The anastomotic site was covered with omentum and returned to the abdominal cavity. The abdominal incision was stitched using standard surgical technique [36].

### GST

Following abdominal incision and exploration of the abdominal cavity, the stomach was exposed and isolated from the abdominal cavity. The incision was made on the body of the stomach (GST) between the greater and lesser curvatures of the least vascularized area of the serosa. Stay sutures were placed on the stomach for easy manipulation of the stomach. The stomach content was emptied, and the incision was closed in two layers of simple continuous and Lembert suture patterns using chromic catgut (size 2-0). The abdominal incisions were stitched with chromic catgut (size 2-0) using simple continuous suture pattern, and the skin incision was stitched with nylon (size 2-0) using horizontal mattress suture pattern.

Blood samples were collected from both cephalic and jugular veins into plain vacutainer tubes for determination of cortisol (CORT), glucose (GLU), total protein (TP), albumin (ALB), globulin (GL), and GL/ALB ratio at 0, 2, 24, 48, 72, 96, 120, and 144 h post-surgical operation.

### Statistical analysis

The data generated were presented as means± standard error of the mean. One-way analysis of variance was used to analyze the data. Turkey’s multiple comparison test was used to detect a significant difference between all the groups at (p<0.05).

## Results

The results of the present study have shown that various surgical procedures have effects on biochemical parameters of the Nigerian local dogs (Table-1). The mean CORT value for animals in Group 1 increased significantly to 257.5±7.9 from 143.8±61.7, 2 h postsurgery (OVH), 168.7±23.1-271.2±6.6 (GST), and 120.9±44.1-278.1±6.5 (IRA), respectively. However, a significant increase in serum CORT level was observed at 96 h; 180.8±34.1 (GST), at 24 h, 214.1±7.0 and 96 h, 228.7±15.2 (IRA), respectively. There was no significant increased CORT level (p>0.05) for the rest of the study period (Table-1).

**Table-1:** Effects of surgical procedures on serum mean CORT.

Surgical procedures	Time (h)							

	0	2	24	48	72	96	120	144
OVH	143.8±61.7	257.5±7.9*	152.7±36.4	168.8±32.9	100.8±6.99	180.8±34.1*	108.5±23.4	130.2±34.4
GST	168.7±23.1	271.2±6.6*	135.6±24.3	204.6±19.1	134.9±22.9	213.6±11.1	207.1±21.2	183.9±17.6
IRA	120.9±44.1	278.1±6.5*	214.1±7.0*	181.4±7.2	163.3±13.4	228.7±15.2*	169.3±11.7	200.6±3.1

* Means are significantly different. OVH=Ovariohysterectomy, IRA=Intestinal resection and anastomosis, GST=Gastrostomy, CORT=Cortisol

Serum GLU level was significantly increased from 49.73±6.5 to 92.05±5.2 (OVH), 72.33±1.2 to 142.6±9.0 (GST), and 52.00±4.1 to 97.48±0.9 (IRA) 2 h postsurgery. There was no significant increase (p>0.05) in GLU level for the rest of the study period (Table-2).

**Table-2:** Effects of surgical procedures on serum mean GLU.

Surgical procedures	Time (h)							

	0	2	24	48	72	96	120	144
OVH	49.73±6.5	92.05±5.2*	50.75±14.7	65.83±8.9	60.90±6.62	54.68±5.2	52.33±2.6	51.23±5.8
GST	72.33±1.2	142.6±9.0*	58.88±8.7	66.33±5.9	68.30±1.8	62.23±1.3	57.15±3.6	26.45±2.7
IRA	52.00±4.1	97.48±0.9*	51.48±15.0	52.48±16.8	59.20±0.5	48.95±14.1	47.38±0.6	70.88±1.0

* Means are significantly different. OVH=Ovariohysterectomy, IRA=Intestinal resection and anastomosis, GST=Gastrotomy, GLU=Glucose

TP was not significantly different (p>0.05) in OVH group. However, TP decreased from 73.70±4.9 before surgery to 54.8±0.7 and 56.35±1.5, 2 and 24 h postsurgery (GST). More so TP was decreased from 83.15±3.9 pre-surgery to 67.85±0.8, 58.78±1.7, 58.28±2.0, 58.10±1.3, 70.10±2.8, and 58.38±0.7 at 2, 24, 72, 96, 120, and 144 h, respectively (Table-3).

**Table-3:** Effects of surgical procedures on serum mean TP.

Surgical procedures	Time (h)							

	0	2	24	48	72	96	120	144
OVH	59.15±2.8	56.23±3.4	68.58±5.5	62.25±2.5	78.58±4.7	72.52±5.1	67.23±6.1	64.55±6.8
GST	73.70±4.9	54.8±0.7*	56.35±1.5*	75.03±0.7	72.75±1.7	77.48±4.2	63.93±0.7	75.08±0.8
IRA	83.15±3.9	67.85±0.8*	58.78±1.7*	75.40±1.6	58.28±2.0*	58.10±1.3*	70.10±2.8*	58.38±0.7*

* Means are significantly different (p<0.05). OVH=Ovariohysterectomy, IRA=Intestinal resection and anastomosis, GST=Gastrotomy, TP=Total protein

The results of various surgical procedures on serum mean ALB are presented in Table-4. There was no statistically significant (p>0.05) increased level of ALB in the OVH and GST group. However, ALB decreased from 40.73±3.7 pre-surgery to 31.45±1.4, 26.60±0.4, 25.18±0.8, 32.10±2.5, and 27.63±0.8 at 2, 24, 72, 96, 120, and 144 h, respectively (Table-4). Furthermore, there was no statistically significant difference (p>0.05) in GL in OVH and GST group during the study. However, GL decreased from 64.60±12.9 before surgery to 45.48±6.9, 45.23±6.5, 45.80±6.5 and 44.88±7.4 at 24, 72, 96, and 144 h post IRA, respectively (Table-5). There was no significant difference in ALB / GL ratio in the three groups (Table-6).

**Table-4:** Effects of surgical procedures on serum mean ALB.

Surgical procedures	Time (h)							

	0	2	24	48	72	96	120	144
OVH	27.63±4.8	24.90±2.7	30.48±4.9	27.45±4.2	33.0±3.5	31.38±2.2	28.6±2.2	25.7±3.8
GST	36.8±4.5	27.7±2.0	28.8±2.4	35.8±2.1	33.8±2.4	35.4±3.2	29.45±1.7	33.80±0.7
IRA	40.73±3.7	31.45±1.4*	26.9±0.3*	36.03±0.3	26.60±0.4*	25.18±0.8*	32.10±2.5*	27.63±0.8*

* Means are significantly different (p<0.05). OVH=Ovariohysterectomy, IRA=Intestinal resection and anastomosis, GST=Gastrotomy, ALB=Albumin

**Table-5:** Effects of surgical procedures on serum mean GL.

Surgical procedures	Time (h)							

	0	2	24	48	72	96	120	144
OVH	31.53±3.3	31.33±6.1	38.15±4.6	34.63±1.6	45.55±5.7	41.15±5.8	38.63±5.8	51.90±6.1
GST	57.73±11.5	42.10±6.9	43.38±7.8	58.15±9.3	54.60±11.1	61.48±11.2	50.23±7.8	58.48±8.8
IRA	64.60±12.9	52.93±8.3	45.48±6.9*	59.85±9.8	45.23±6.5*	45.80±6.5*	55.40±10.0	44.88±7.4*

* Means are significantly different (p<0.05). OVH=Ovariohysterectomy, IRA=Intestinal resection and anastomosis, GST=Gastrotomy, GL=Glucose

**Table-6:** Effects of surgical procedures on serum mean ALB/GL ratio.

Surgical procedures	Mean±SEM (h)							

	0	2	24	48	72	96	120	144
OVH	0.88±1.5	0.79±0.4	0.79±1.1	0.79±2.6	0.72±0.6	0.76±0.4	0.74±0.4	0.49±0.6
GST	0.64±0.4	0.65±0.3	0.66±0.3	0.62±0.2	0.62±0.2	0.58±0.3	0.59±0.2	0.58±0.0
IRA	0.63±0.3	0.59±0.2	0.59±0.0	0.60±0.0	0.59±0.1	0.55±0.1	0.58±0.3	0.62±0.1

OVH=Ovariohysterectomy, IRA=Intestinal resection and anastomosis, GST=Gastrotomy, SEM=Standard error of the mean, ALB=Albumin, GL=Glucose

## Discussion

The increased CORT level is in agreement with the findings of [37], indicating that CORT concentration could be suppressed 30 min after induction of West African Dwarf goats with xylazine and propofol and a surge in CORT concentrations was observed 2 h postsurgery. Similar findings were reported postsurgery [33,38]. The value of CORT began to fall at 24 h postsurgery for the three groups and was significant at 24 h for Group 3 and 96 h for both Groups 1 and 3. The findings suggest that IRA is more traumatic than OVH and GST.

GLU concentrations were significantly high at 2 h postsurgery coinciding with peak CORT levels because CORT stimulates GLU production, decreased peripheral use of GLU and increased GLU concentrations which are in turn proportional to the degree of surgical trauma [14,39].

Surgery and trauma have been reported to decrease protein synthesis and increase protein catabolism [40], and these changes are associated with the level and duration of trauma [16]. Catabolism caused a decline in protein synthesis and an increase in amino acid oxidation within the first 2 h following surgery, in patients who underwent colectomy [41]. The decreased protein values in the present study suggest that IRA causes more trauma than OVH and GST. The intestinal lesions created in IRA also contributed to protein loss [42]. Assessment of ALB in 2 h disagrees with the findings that ALB can only be assessed in 4-6 h [43]. ALB shows an immediate response to surgical stress and as such is used to measure surgical stress [44-46]. Furthermore, postoperative ALB decrease in proportion to the magnitude of surgery may be used as a predictor for a complicated postoperative course. Surgery, sepsis, and burn affects protein metabolism [47,48], and evaluation of ALB will provide reliable information on the magnitude of the trauma [44,48,49].

The production of acute phase proteins during the early postoperative phase, increased basal energy expenditure and consumption of significant proportion of the body’s store of protein to pave the way for glyconeogenesis are the cause of hypoalbuminemia during trauma [50]. Capillary escape of ALB is responsible for the postoperative loss of ALB [51,52] and can be up to100% after major surgery and 300% in septic shock [44]. Concurrent decrease ALB and GL concentration may be due to inflammation that led to the production of immuno GLs and acute phase reacting proteins [48].

## Conclusion

CORT, GLU, TP, and ALB are good biomarkers of surgical stress response in the xylazine-propofol anesthetic protocol for GST, IRA, and OVH.

## Authors’ Contributions

AIK designed the research. TT, ATE, and AIK carried out the surgery. AIK and TT wrote the manuscript. ATE revised the manuscript. All authors read and approved the final manuscript.
